# Adenocarcinoma arising from widespread heterotopic gastric mucosa in the cervicothoracic esophagus: a case report

**DOI:** 10.1186/s40792-023-01707-7

**Published:** 2023-07-20

**Authors:** Shohei Nogi, Kazuhiro Noma, Masashi Hashimoto, Takuya Kato, Naoaki Maeda, Shunsuke Tanabe, Yasuhiro Shirakawa, Toshiyoshi Fujiwara

**Affiliations:** 1 Department of Gastroenterological Surgery, Okayama University Graduate School of Medicine, Dentistry and Pharmaceutical Sciences, 2-5-1 Shikata-Cho, Kita-Ku, Okayama, 700-8558 Japan; 2 Department of Surgery, Hiroshima City Hiroshima Citizens Hospital, 7-33 Motomachi, Naka-Ku, Hiroshima, Japan

**Keywords:** Heterotopic gastric mucosa, Esophagus, Adenocarcinoma

## Abstract

**Background:**

In Japan, about 6% of esophageal cancers are adenocarcinomas, although most of them arise from Barrett’s epithelium. Adenocarcinoma arising from heterotopic gastric mucosa (HGM) is very rare. Due to its rarity, there is no unified view on its treatment strategy and prognosis.

**Case presentation:**

A 57-year-old man presented with a protruding lesion in the cervicothoracic esophagus that was detected by an upper gastrointestinal series at a medical checkup. Esophagoscopy revealed a 30 mm Type 1 tumor circumferentially surrounded by widespread HGM. Computed tomography (CT) and fluorodeoxyglucose (FDG) positron emission tomography (PET)/CT showed no metastasis or invasion of the surrounding organs. We diagnosed the lesion as cT2N0M0 cStageIIB [Union for International Cancer Control (UICC) 8th Ed] cancer and performed subtotal esophagectomy with three-field lymph node dissection. The tumor was determined to be a well-differentiated adenocarcinoma arising from HGM, with deep invasion of the submucosa. The patient underwent no adjuvant therapy and has currently survived without any evidence of recurrence for 15 months.

**Conclusions:**

Although the treatment for adenocarcinoma arising from HGM is basically the same as that for squamous cell carcinoma (SCC) of the esophagus, it is important to determine the treatment strategy based on the characteristics of the adenocarcinoma arising from HGM.

## Background

Heterotopic gastric mucosa (HGM) is defined by the presence of gastric mucosal tissue outside the stomach. It can occur anywhere in the gastrointestinal tract. HGM in the esophagus is regarded as a congenital developmental anomaly resulting from an incomplete embryological esophageal epithelialization process.

In Japan, about 6% of esophageal cancers are adenocarcinomas [1], with most of them arising from Barrett’s epithelium, which is an acquired intestinal metaplasia secondary to gastroesophageal reflux disease. Adenocarcinomas arising from HGM are, however, rare. Due to their rarity, there is no unified view on the treatment strategy and prognosis of adenocarcinomas arising from HGM.

Here, we report a case of esophageal adenocarcinoma arising from HGM that was surgically treated, and suggest possible treatment strategies based on a literature review.

## Case presentation

A 57-year-old man presented with a protruding lesion in the cervicothoracic esophagus that was detected by an upper gastrointestinal series at a medical checkup. His medical history was unremarkable except for hypertension. His family history was relevant for thyroid cancer in his daughter. He was an inveterate drinker and smoker, drinking 1.4 standard drinks of beer per day and smoking 20 cigarettes per day for 30 years.

**Fig. 1:**
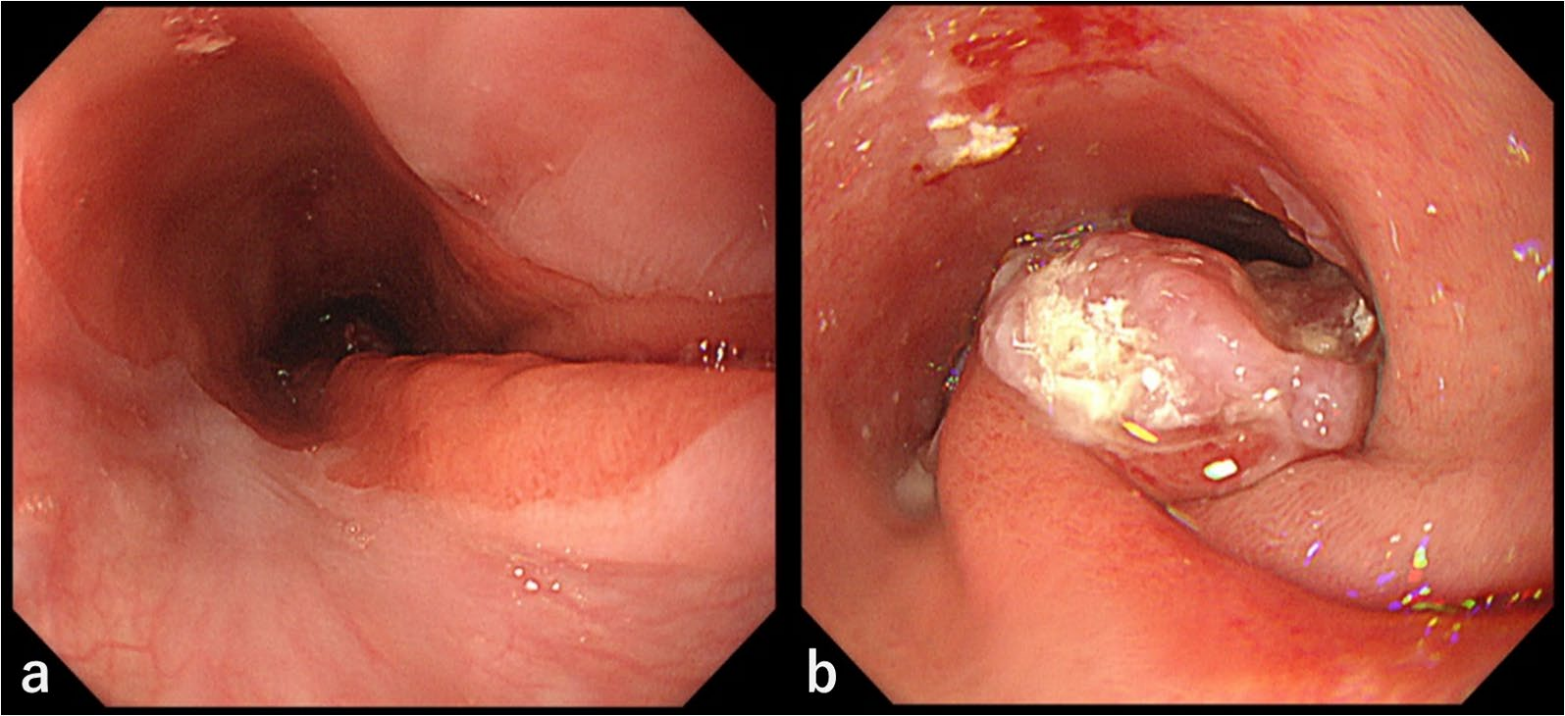
Esophagoscopy findings. **a** Widespread HGM extended from 16 to 24 cm distal to the incisor teeth. **b** Type 1 tumor circumferentially surrounded by HGM

**Fig. 2:**
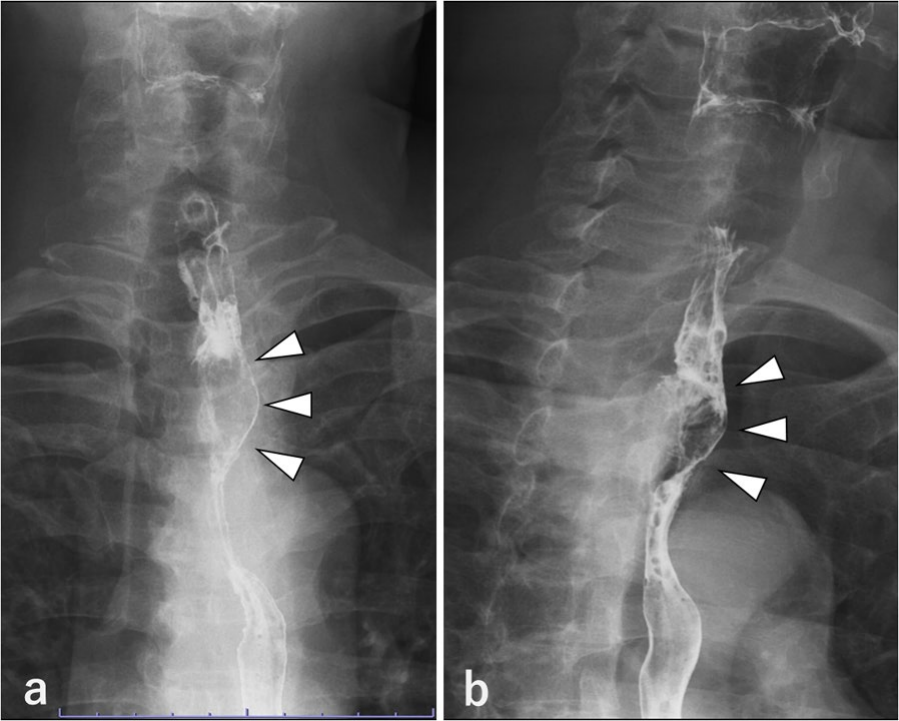
Barium esophagography images. Front view (**a**) and left oblique view (**b**) showing an irregular elevated lesion in the posterior wall of the cervicothoracic esophagus (arrowhead)

**Fig. 3:**
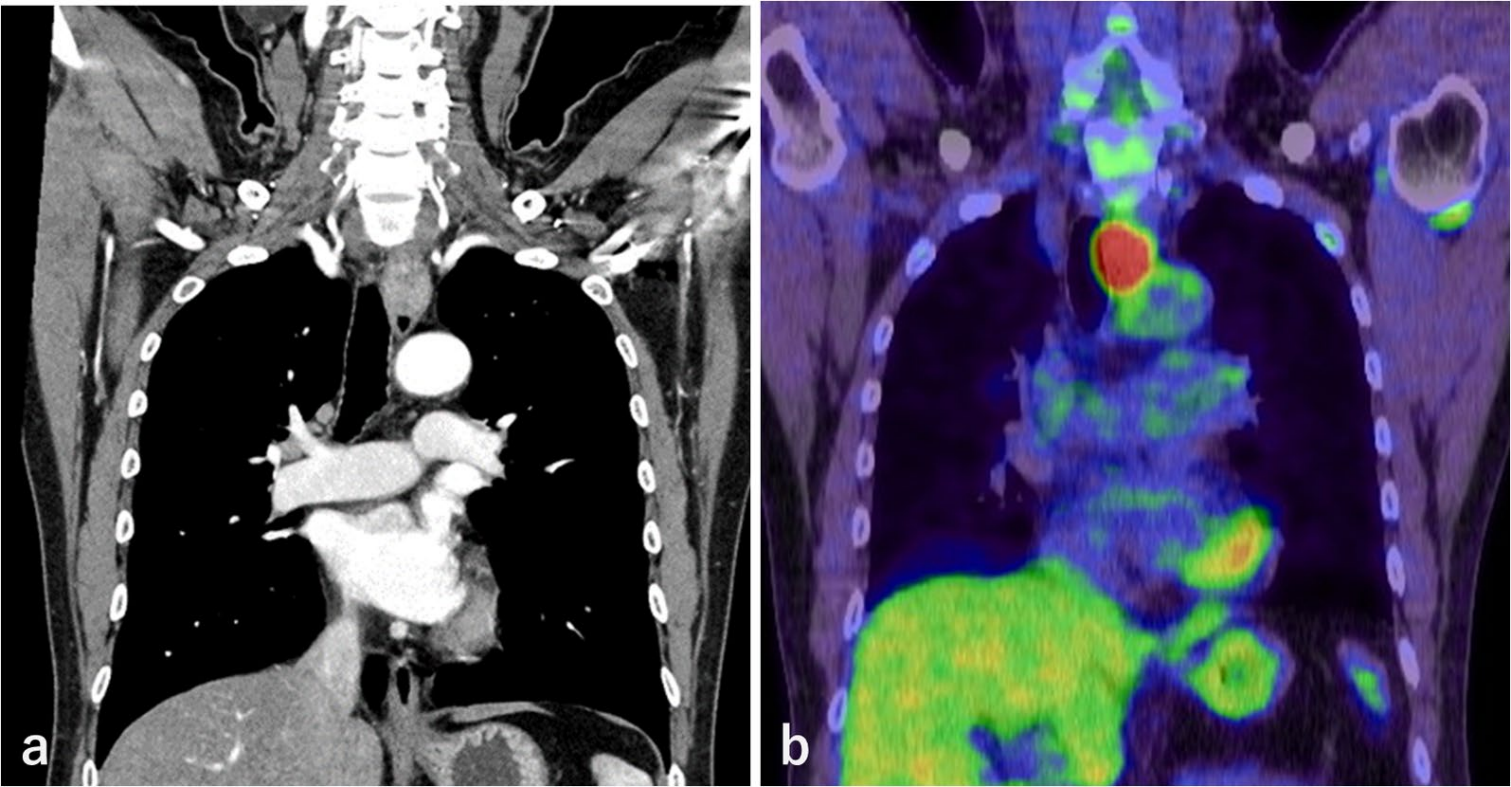
Contrast-enhanced CT and FDG–PET/CT images. **a** Contrast-enhanced CT showed enhanced wall thickening in the cervicothoracic esophagus that displaced the trachea to the right side. **b** FDG–PET/CT showed high FDG uptake with a maximum SUV of 21.6 only in the primary lesion

Blood tests showed no remarkable findings, and the levels of tumor markers, such as carcinoembryonic antigen (CEA), carbohydrate antigen 19-9 (CA19-9), and squamous cell carcinoma (SCC) antigen, were all within the standard values. Esophagoscopy revealed a 30 mm Type 1 tumor in the posterior wall at 20 cm to 23 cm distal to the incisors, which was circumferentially surrounded by widespread HGM. The HGM extended from 16 to 24 cm distal to the incisors (Fig. 1a, b). Biopsy specimens from the tumor revealed adenocarcinoma. We applied a marking clip on the oral edge of the HGM at the time of esophagoscopy. Barium esophagography showed an irregular elevated lesion on the posterior wall of the cervicothoracic esophagus (Fig. 2a, b). Contrast-enhanced computed tomography (CT) of the neck, chest and abdomen showed enhanced wall thickening in the cervicothoracic esophagus, that displaced the trachea to the right side, although there was no obvious invasion (Fig. 3a). Fluorodeoxyglucose (FDG) positron emission tomography (PET)/CT showed high FDG uptake with a maximum standardized uptake value (SUV) of 21.6 only in the primary lesion (Fig. 3b). The tumor was diagnosed clinically as T2, N0, M0, Stage IIB adenocarcinoma in the cervicothoracic esophagus [Union for International Cancer Control (UICC) 8th Ed].

The patient underwent thoracoscopic subtotal esophagectomy with three-field lymph node dissection and gastric tube reconstruction by the posterior mediastinal route. Intraoperative evaluation revealed that the marking clip on the oral edge of the HGM was at the esophageal orifice. Before anastomosis, we evaluated blood flow in the gastric tube by fluorescence angiography using indocyanine green, and found poor blood flow at the tip of the gastric tube. Since anastomosis at the esophageal orifice was extremely risky, we performed anastomosis at the site, where the HGM partially remained. We also confirmed the absence of malignancy in the oral margins of the resected specimen by intraoperative frozen section analysis.

**Fig. 4:**
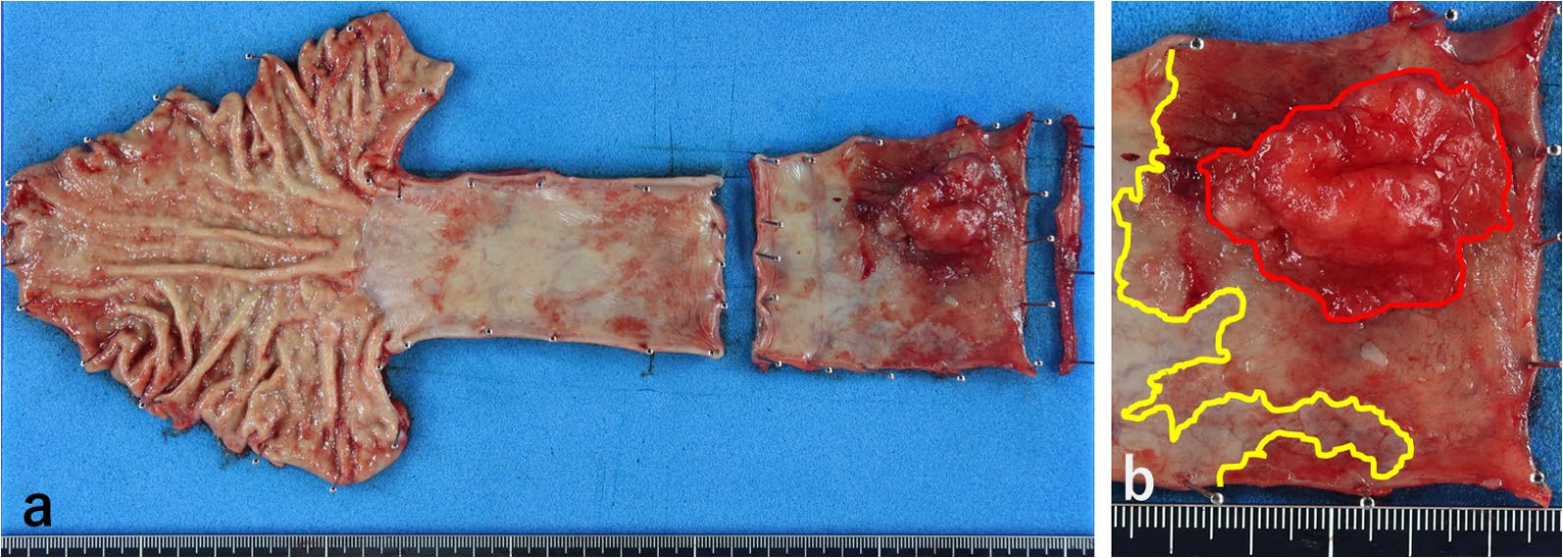
Resected esophageal carcinoma specimen. **a** Gross evaluation showed a type 0-I + 0-IIa tumor, 43 × 40 mm in the cervicothoracic esophagus. **b** Enlarged view showing the salmon colored mucosa (yellow line) circumferentially surrounding the tumor (red line)

**Fig. 5:**
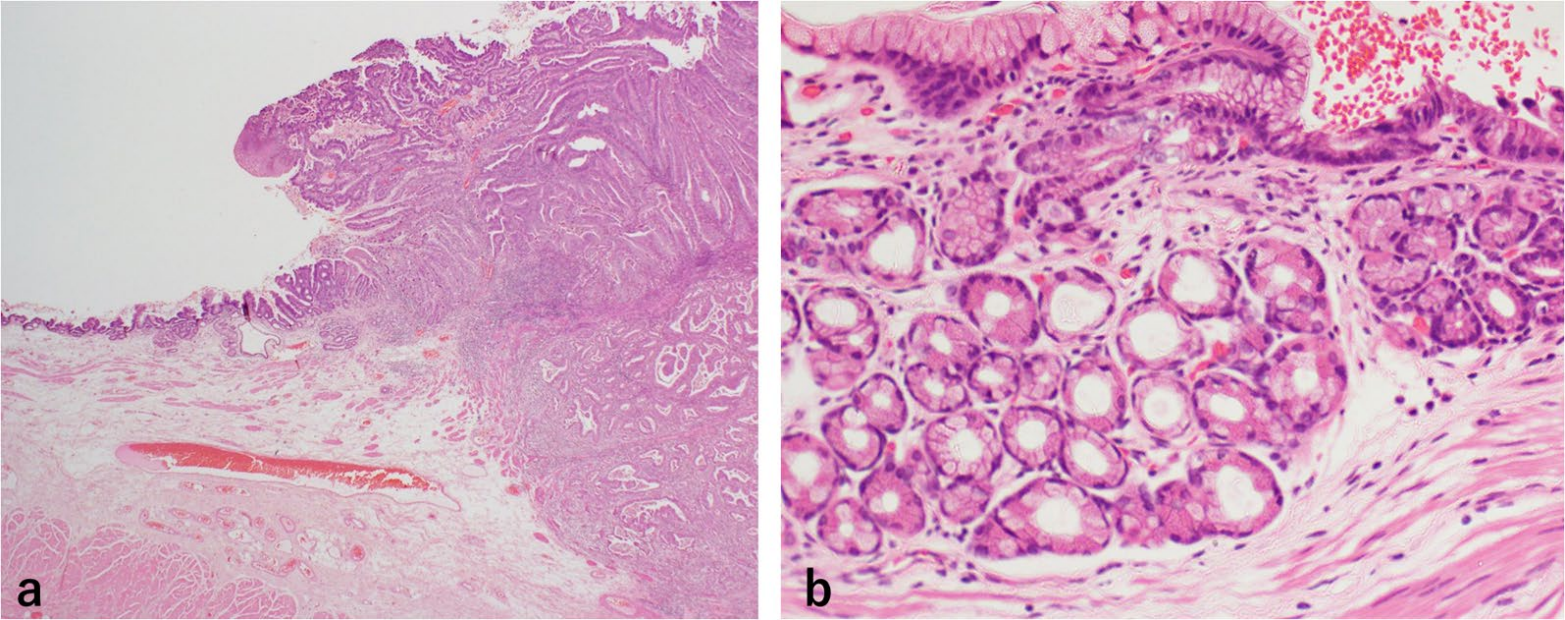
Pathological evaluation of the resected specimen. **a** Hematoxylin and eosin (H.E.) staining showed a well-differentiated tubular adenocarcinoma infiltrating deep into the submucosa. **b** Microscopic evaluation showed grades of HGM without atypia, and intraepithelial carcinoma. The HGM without atypia contained gastric fundic glands with parietal cells without intestinal metaplasia or *Helicobacter pylori* infection

**Fig. 6:**
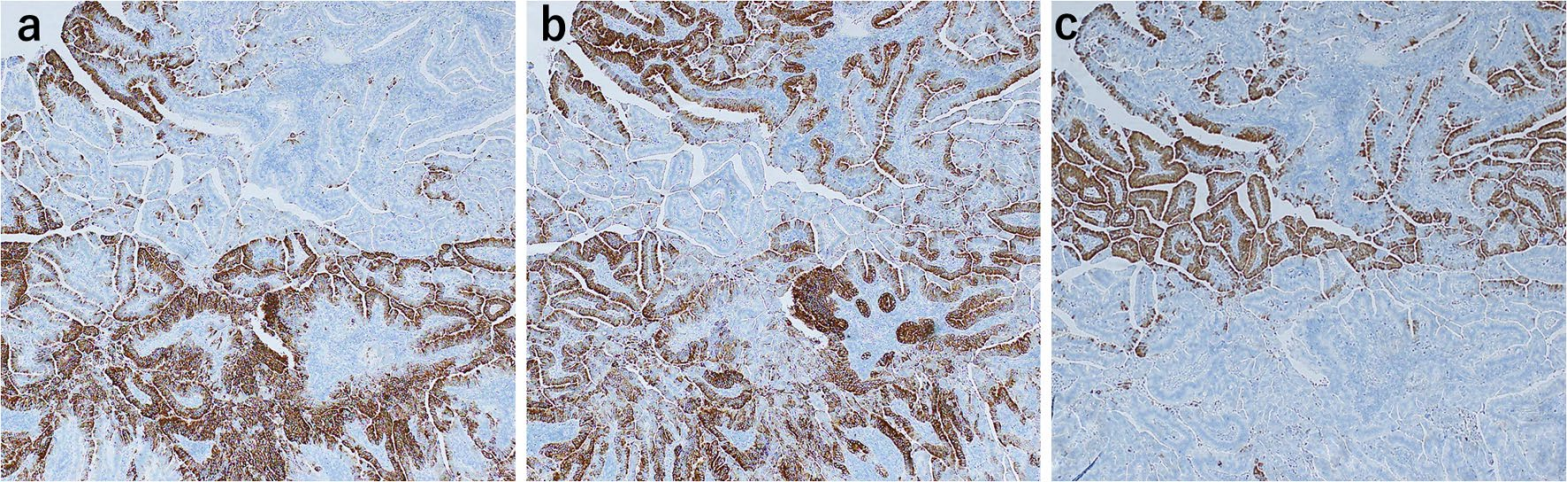
Results of immunohistochemical analysis. **a** MUC5AC staining was positive mainly in the invasive area. **b** MUC6 staining was positive mainly in the invasive area, as with MUC5AC. **c** MUC2 was partially positive in the surface layer of the tumor

The resected specimen contained an approximate 17 cm length of the esophagus. The tumor was a sharply demarcated, 43 × 40 mm elevated lesion (Type 0-I + 0-IIa). Salmon-colored mucosa circumferentially surrounded the tumor (Fig. 4a, b). Microscopic evaluation showed grades of HGM without atypia, intraepithelial carcinoma, and invasive well-differentiated tubular adenocarcinoma. The tumor infiltrated deep into the submucosa, but not into the muscularis propria (Fig. 5a, b). Immunohistochemical analysis showed that the tumor was positive for MUC5AC and MUC6, with scattered MUC2-positive cells, indicating a mixed gastrointestinal phenotype with a predominance of gastric type phenotype (Fig. 6a–c). On the other hand, the HGM without atypia contained gastric fundic glands with parietal cells, was negative for MUC2, and showed no intestinal metaplasia or *Helicobacter pylori* infection. The regional lymph nodes were negative for metastasis.

Based on these findings, we made a diagnosis of well-differentiated adenocarcinoma (Type 0-I + 0-IIa, pT1b-SM, N0, M0, pStage IB [UICC 8th Ed]. Furthermore, the pathologist determined that there was lymphatic invasion (ly1, v0) by hematoxylin–eosin staining, without immunohistostaining. The patient’s postoperative course was uneventful, although he was transferred to the hospital on the 25th day after surgery due to inadequate oral intake. As of today, 15 months after surgery, no signs of recurrence have been observed despite no adjuvant therapy.

## Discussion

Advances in endoscopic equipment and diagnostic capabilities have led to the more frequent detection of HGM of the esophagus in recent years. The reported prevalence rate of HGM is 11–14.5% [2, 3]. HGM usually has no clinical significance, although when parietal cells exist, their acid secretion might cause chronic inflammation and ulceration, manifesting clinically as dysphagia and pharyngeal discomfort [4]. The width of HGM usually ranges from 3 to 30 mm, and it is categorized as small (< 15 mm) or large (> 15 mm)-sized HGM based on the width of the HGM [5]. In this case, the circumferential HGM extending 8 cm in the long axis was much larger than previously reported, which seems to be extremely rare. Since adenocarcinomas arising from HGMs are extremely rare compared to the incidence of HGM, the malignant potential of HGM seems to be low [6]. However, since there have been previous cases of adenocarcinoma arising from widespread HGM extending for more than 3 cm [7–9], cases with widespread HGM require cautious follow-up.

Tanaka et al. previously reported the possibility that intestinal metaplasia of HGM contributes to carcinogenesis, as in gastric adenocarcinoma [10]. However, our patient did not have evidence of *H. pylori* infection, and immunohistochemical analysis did not reveal intestinal metaplasia. On the other hand, since the HGM in this case contained gastric fundic glands with parietal cells, chronic inflammation due to acid secretion might have contributed to carcinogenesis, as reported by Satoh et al. [11]. Akanuma et al. reported 43 cases of adenocarcinoma arising from HGM of the esophagus [12]. Of them, 88.4% of the patients were male, and their median age was 60.4 years, which is relatively young. Another feature of adenocarcinoma secondary to HGM is that it is predominantly located in the cervical esophagus, and about half of the cases develop as an elevated growth. Patients might be found incidentally without symptoms or might experience dysphagia due to tumor growth. The epidemiology of this case was consistent with previous reports.

In terms of diagnosis, it is difficult to observe the esophageal orifice with an endoscope. Therefore, if the tumor is large, estimating the depth of the lesion by endoscopy is often difficult. In such cases, endoscopic ultrasonography might be useful in diagnosing the depth of the lesion [13]. We often experience difficulty in distinguishing wall thickening in early-stage lesions from that in advanced cancers even with contrast-enhanced CT. Therefore, we performed a PubMed search using the search terms esophagus, adenocarcinoma and heterotopic gastric mucosa, inlet patch, ectopic and gastric mucosa, evaluating only English articles. We identified 16 previous cases with a clear preoperative diagnosis and upfront resection, including our case [7–10, 14–23] (Table 1).

**Table 1:** Review of esophageal adenocarcinomas arising from HGM with a clear preoperative diagnosis and upfront resection

First author	Years	Localization	Age	Sex	Tumor type	Tumor size (mm)	cT	pT	Treatment
Klaase [14]	2001	CeUt	43	M	Unclear	40	3	4b(trachea)	Laryngopharyngoesophagectomy
Pech [7]	2001	Ut	77	M	0-II	3	1a	1a-MM	Endoscopic mucosal resection
Hirayama [15]	2003	Ce	77	F	0-Ip	21	1a	1a-LPM	Endoscopic mucosal resection
Komori [16]	2010	Ut	75	M	1	35	2	1a	Esophagectomy
Yoshida [17]	2010	Ce	79	M	0-I	4	1	is-EP	Endoscopic mucosal resection
Tanaka [10]	2014	Ce	70s	M	0-IIc	25	1a	1a-MM	Partial resection
Yasar [18]	2014	Ce	52	F	0-I	7	1a	1a	Endoscopic mucosal resection
Ajmal [19]	2015	Ce or Ut	57	M	1	30	2	1b	Esophagectomy
Kadota [20] Case1	2016	Ce	78	F	0-Is + IIc	19	1a	1b	Endoscopic submucosal dissection
Case 2	2016	Ut	70	M	2	42	3	3	Laryngopharyngoesophagectomy
Hudspeth [21]	2016	Ce or Ut	77	M	0-I	15	1a	1a	Endoscopic submucosal dissection
Gushima [22]	2017	Ce	65	F	0-I	16	1	1b	Endoscopic submucosal dissection
Oono [8]	2019	Ce	58	M	0-Is	32	1a	1a-MM	Endoscopic submucosal dissection
Ohki [9]	2022	Ce or Ut	59	M	0-I + IIa	44	1a	1a-MM	Endoscopic submucosal dissection
Kitasaki [23]	2022	UtCe	52	M	0-Ip	20	1a	1a-MM	Endoscopic submucosal dissection
Our case	2022	UtCe	57	M	1	43	2	1b	Esophagectomy

Of the five cases preoperatively diagnosed as advanced cancer, three cases were found to have superficial cancer by pathological evaluation. On the other hand, none of the 11 cases diagnosed as superficial carcinoma were found to have advanced carcinoma by pathological evaluation. This suggests that heterotopic gastric mucosal carcinoma tends to be overestimated preoperatively.

Since adenocarcinoma arising from HGM is extremely rare, there is no unified view on its treatment strategy and prognosis. However, since HGM is a remnant of fetal columnar epithelium, the structure of the submucosa and deeper tissue is the same as that of the normal esophagus. Therefore, we assumed that the risk of metastasis in cases of submucosal invasion would be similar to that of typical esophageal cancer. In fact, Nomura et al. reported that two of nine cases of adenocarcinoma arising from HGM with submucosal invasion were positive for lymph node metastasis, suggesting that lymphatic invasion and lymph node metastasis might occur even in early cancer stages [24]. In view of the above, we chose to perform subtotal esophagectomy with three field lymph node dissection, because we thought that treatment similar to that for the usual esophageal cancer was necessary in this case. Although we also considered administering neoadjuvant chemotherapy, since we estimated that the patient had advanced cancer, we preferred upfront surgery for two reasons: first, the patient had no obvious metastasis and the invasion depth was unclear; and second, if the tumor progressed despite chemotherapy, we would have had to perform pharyngolaryngoesophagectomy, which would significantly impair the patient’s quality of life. Subsequently, since pathological analysis revealed T1b-SM, N0 cancer, adjuvant therapy was not considered necessary. Our experience suggests that in cases without obvious metastasis, the treatment strategy of upfront surgery followed by consideration of adjuvant therapy based on the pathological diagnosis might be effective.

In this case, we retained some of the HGM in situ, based on the intraoperative findings. To the best of our knowledge, there is only one report of metachronous carcinogenesis from the HGM remaining after endoscopic submucosal dissection [23]. Although complete resection of the HGM is desirable, particularly when it is associated with considerable risk, such as due to poor blood flow at the reconstructed organ, incomplete resection is an acceptable option under the premise of strict postoperative follow-up.

## Conclusions

We performed curative surgery for a rare adenocarcinoma arising from widespread HGM of the esophagus. Although we treated the patient according to the treatment protocol for esophageal squamous cell carcinoma, it is important to consider the characteristics of adenocarcinoma arising from HGM when determining the treatment strategy. Particularly, when HGM exceeds 3 cm, special attention should be given to the potential development of adenocarcinoma.

## Data Availability

All the data pertaining to this case report has been presented in the article.

## Abbreviations

HGM: Heterotopic gastric mucosa
CT: Computed tomography
FDG: Fluorodeoxyglucose
PET: Positron emission tomography
UICC: Union for International Cancer Control
SCC: Squamous cell carcinoma
CEA: Carcinoembryonic antigen
CA19-9: Carbohydrate antigen 19-9
SUV: Standardized uptake value
